# Supplementary material for: A cortical pool of LIN-5 (NuMA) controls cytokinetic furrow formation and cytokinesis completion
Source: J Cell Biol. 2025 Apr 30;224(7):e202406059. doi: 10.1083/jcb.202406059 (PMC12042773; doi:10.1083/jcb.202406059)
Supplement: Table S1 — lists C. elegans strains. [file jcb_202406059_tables1.docx]

**Table S1**

| **Strains** | **Genotype** | **Source** |
| --- | --- | --- |
| SA250 | tjIs54 [pie-1p::GFP::tbb-2 + pie-1p::2xmCherry::tbg-1 + unc-119(+)]. tjIs57 [pie-1p::mCherry::his-48 + unc-119(+)] | CGC |
| SK008 | tjIs54 [pie-1p::GFP::tbb-2 + pie-1p::2xmCherry::tbg-1 + unc-119(+)]. tjIs57 [pie-1p::mCherry::his-48 + unc-119(+)] ;GFP:PH^PLC1δ1^, the PH domain (aa 1–175) from PLC1δ1 (accession no. P10688) (Audhya et al., 2005); | This study |
| ﻿  SK009 | GFP:PH^PLC1δ1^, the PH domain (aa 1–175) from PLC1δ1 (accession no. P10688) (Audhya et al., 2005); cherry-tubulin (Kapoor and Kotak, 2019) | This study |
| ﻿  LP585 | lin-5::mNG- lin-5(cp288[lin-5::mNG-C1^3xFlag]) II | CGC |
| ﻿  LP162 | nmy-2(cp13[nmy-2::GFP + LoxP])I. | CGC |
| ﻿  OD4337 | zen-4-Scarlet ﻿*C. elegans*: Strain OD4338: ltSi1491[pSG121; Pzen-4::zen-4 RE-encoded-exon6::Scarlet::zen-4 3’-UTR; cb unc-119(+)]I; ltSi1480 [pSG113; ﻿unc-119(ed3)III | Gómez-Cavazos et.al., 2020 |
| ﻿  OD4077 | mNG::ECT-2- ﻿ltSi1480 [pSG113; Pect-2::mNeonGreen::ect-2 RE-encoded- exon8::ect-2 3’-UTR; cb unc-119(+)]II;unc-119(ed3)III | Gómez-Cavazos et.al., 2020 |
| ﻿  OD3619 | cyk-4-mNG ﻿ltSi1124 [pSG092; Pcyk-4::CYK-4reencoded::mNeongreen::cyk-4::cyk-4 3’-UTR; cb unc-119(+)]II; unc-119(ed3)IIIC. | Gómez-Cavazos et.al., 2020 |
| ﻿  SV124 | lin-5 (ev571)I | CGC |
| ﻿ WH12 | spd-1(oj5)I | CGC |
| SK010 | tjIs54 [pie-1p::GFP::tbb-2 + pie-1p::2xmCherry::tbg-1 + unc-119(+)]. tjIs57 [pie-1p::mCherry::his-48 + unc-119(+)] ;GFP:PH^PLC1δ1^; spd-1(oj5)I | This study |
| SK011 | cyk-4-mNG ﻿ltSi1124 [pSG092; Pcyk-4::CYK-4reencoded::mNeongreen::cyk-4::cyk-4 3’-UTR; cb unc-119(+)]II; unc-119(ed3)IIIC; spd-1(oj5)I | This study |
| SK012 | nmy-2(cp13[nmy-2::GFP + LoxP]) I.; lin-5 (ev571)II | This study |
| SK013 | GFP:PH^PLC1δ1^, the PH domain (aa 1–175) from PLC1δ1; cherry-tubulin; lin-5 (ev571)II | This study |
| SK026 | 327-GFP nmy-2 integrated; life-act; tjIs54 [pie-1p::GFP::tbb-2 + pie-1p::2xmCherry::tbg-1 + unc-119(+)] | This study |
| ZAN351 | ﻿  nmy-2(cp52[nmy-2::mkate2 + LoxP unc-119(+) LoxP]) I; unc-119(ed3) III; ani-1(mon7[mNeonGreen^3xFlag::ani-1]) III | Lebedev et al., 2023 |
| SK030 | tjIs54 [pie-1p::GFP::tbb-2 + pie-1p::2xmCherry::tbg-1 + unc-119(+)].;spd-1(oj5)I | This study |
| SK031 | ani-1(mon7[mNeonGreen^3xFlag::ani-1]) III; spd-1(oj5)I | This study |
