# Supplementary material for: A cortical pool of LIN-5 (NuMA) controls cytokinetic furrow formation and cytokinesis completion
Source: J Cell Biol. 2025 Apr 30;224(7):e202406059. doi: 10.1083/jcb.202406059 (PMC12042773; doi:10.1083/jcb.202406059)
Supplement: Table S2 — shows bacterial feeding strains and primers used to design RNAi clone. [file jcb_202406059_tables2.docx]

**Table S2- ﻿Bacterial feeding strains and primers used to design RNAi clone**

| **﻿RNAi against** | **RNAi libraries [VL (Vidal Lab; Rual et al., 2004)* and AR (Ahringer Lab; Kamath et al., 2003)*]** | **RNAi condition** |
| --- | --- | --- |
| ﻿*lin-5* | AR (T09A5.10/ II/ E05); Cloned in L4440 | 24-60 h |
| *﻿hcp-4* | VL (T03F1.9/11023/G8) | 48-60 h |
| *﻿zyg-9* | AR (F22B5.7/ II/ G06) | 24 hours |
| *﻿par-2* | Cloned in L4440 (source: Motegi) | 48-60 h |
| *﻿par-3* | AR (F54E7.3/ III/ A01) | 48-60 h |
| *﻿par-5* | VL (M117.2/10018/F4) | 48 h |
| *gpr-1* | VL (F22B7.13/11008/B4) | 60-70 h |
| *gpr-2* | VL (C38C10.4/11008/C3) | 60-70 h |
| *aspm-1* | Cloned in L4440 | 48 h |
| *zen-4* | VL (M03D4.1/10015/ D7) | 48 h |
| **Primers for RNAi** | **Enzyme site** | **Sequence (5’-3’)** |
| aspm-1 forward primer | aspm-1 with NotI site | gcGCGGCCGCATGGATAATAACGTTGAGGAT |
| aspm-1 reverse primer | aspm-1 with HindIII site | gcAAGCTTTTCCAAAAGTGATGG |
| lin-5 forward primer | Lin-5 with NotI site | gcGCGGCCGCATGAGCGTGAGCACATCAG |
| lin-5 reverse primer | lin-5 with BamH1 site | gcGGATCCAGGGGAATCGACAGCCTCC |
